# Editorial: Allergen source-specific mucosal barrier disruptors

**DOI:** 10.3389/falgy.2024.1466954

**Published:** 2024-08-12

**Authors:** Rajna Minić, Lidija Burazer, Andrijana Nešić, Sabine Flicker

**Affiliations:** ^1^ Institute of Virology, Vaccines and Sera “Torlak”, Belgrade, Serbia; ^2^ Department of Biochemistry, Faculty of Chemistry, University of Belgrade, Belgrade, Serbia; ^3^ Institute of Pathophysiology and Allergy Research, Center for Pathophysiology, Infectiology and Immunology, Medical University of Vienna, Vienna, Austria

**Keywords:** mucosal barrier, barrier hypothesis, mucosal barrier disruptors, functional dyspepsia, epithelial–mesenchymal transition, deoxynivalenol

**Editorial on the Research Topic**
Allergen source-specific mucosal barrier disruptors

The rise of allergic diseases has concerned medical doctors and scientists for years, and several hypotheses on the causation have been presented. The “hygiene hypothesis” (1) was the first theory to explain the increase in the prevalence of allergies, stating that the lack of diverse microbial stimulation contributes to the growing incidence of allergic disorders. Yet, of greater significance to this topic is the “barrier hypothesis,” coined by Pothoven and Schleimer (2) in 2017, and the “extended epithelial barrier hypothesis” presented by Cezmi Akdiş in 2021 (3). Akdiş lecture, during the ECI 2021 congress raised concern among participants about the future trend. Namely, the “extended epithelial barrier hypothesis” states that the massive introduction of surface-active molecules abundant in hygiene products, detergents, and cleaners can damage the epithelial barrier, leading to allergen penetration. However, this loss of barrier integrity not only triggers allergic sensitization but also affects the clinical course of autoimmune and neuropsychiatric diseases.

The translation of this hypothesis to the mechanisms of “classical” allergenicity implies that natural mucosal barrier disruptors are also present in conventional allergen sources, causing similar injury to the epithelial barrier. Among these allergenic sources, allergens with proteolytic activity are considered the most notorious (4). However, there is limited research on the involvement of lipids and other hydrophobic molecules in pollen sensitization (5, 6).

This Special Research Topic offers a comprehensive picture of the current research focusing on allergen source-specific mucosal barrier disruptors and beyond.

The five articles published within this Special Research Topic are quite diverse covering the subject from different points of view. The review by Parron-Ballesteros et al. offered a carefully prepared global view of the epithelial barrier-damaging exposomes impairing the airways and the intestinal compartment. Special focus was given on the microbiota composition, its role as producers of short-chain fatty acids, powerful anti-inflammatory agents, and the gut–lung axis, a hot topic influencing the clinical outcome not only of allergic diseases.

Raith and Swoboda presented an exquisitely written review focused on birch pollen and birch pollen allergy. Current knowledge about internal and external influences impacting the allergenicity of birch pollen and its interactions with the epithelial barrier shows that there are still several challenges that need to be addressed. Furthermore, this contribution raises the awareness that the recombinant Bet v 1 alone might not be the best surrogate when studying the influence of birch pollen on the integrity of airway epithelial cells. The authors conclude that a combination of different approaches is required, such as cell culture models and patient studies, to uncover the molecular pathology and hopefully bring more effective treatments and prevention measures.

Epidermal barrier dysfunction is also an important aspect of atopic dermatitis pathogenesis, yet disease management is currently limited to topical corticosteroids to alleviate symptoms. Further insights into the pathogenesis are needed to reveal novel treatment options. One innovative approach to support tissue repair is the application of epithelial cells that can transform into a migratory mesenchymal phenotype, in a process known as the epithelial–mesenchymal transition (EMT). Although EMT has been highlighted as a possible mechanism for the remodeling process in asthma, the literature investigating atopic dermatitis and EMT is scarce. In their actual review article, Anderson et al. focused on this knowledge gap and identified the induction of the EMT pathway as a promising therapeutical target for atopic dermatitis and the inhibition of the same pathway in the treatment of asthma.

Moreover, a dysregulated epithelial barrier plays an important role in functional dyspepsia, a gastrointestinal disorder. Within this topic, Hari et al. conducted a broad PubMed search for their narrative review to finally present a hypothesis on the involvement of hypoxia-inducible factors (HIFs) in functional dyspepsia. What is especially interesting about this condition is that it occurs with high frequency ranging from 7.2% (7) to 16% (8) and that 34% of functional dyspepsia patients suffer from a psychiatric condition (9), highlighting the strength of the gut–brain interaction, but what is even more interesting is that although sounding pretty straightforward, the pathogenesis on the molecular level is still elusive.

As a highlight of this topic, Drønen et al. provided their original research which, through a series of well-planned and executed experiments, pinpointed the known mucosal barrier disruptor deoxynivalenol (DON), a trichothecene mycotoxin produced by common cereal crops fungal contaminants (10), as an adjuvant in a peanut allergy model. DON also has a more prominent effect in comparison to the cholera toxin (CT), a known activator of adenylate cyclase-cAMP system (11) on early allergy markers such as IL-33 and thymic stromal lymphopoietin (TSLP) levels in the intestines and ST2 levels in serum, showing that different kinds of mucosal barrier disruptors, such as DON and CT, exert different activities at the early stages of peanut sensitization.

The results from this study clearly show that the increase in epithelial barrier-damaging agents linked to industrialization and modern lifestyle contribute to the rise in allergic disorders.

The collection of data within this Special Research Topic demonstrates that maintenance and restoration of the epithelial barrier integrity is a crucial factor affecting various underlying pathways in allergic, autoimmune, and other chronic conditions.

Gaining a deeper understanding of the consequences of harmful environmental factors on the disruption of the epithelial barrier will hopefully sensitize policymakers and individuals to prioritize less toxic alternatives to stop the progress of inflammatory disorders.
